# Analyzing small data sets using Bayesian estimation: the case of posttraumatic stress symptoms following mechanical ventilation in burn survivors

**DOI:** 10.3402/ejpt.v6.25216

**Published:** 2015-03-11

**Authors:** Rens van de Schoot, Joris J. Broere, Koen H. Perryck, Mariëlle Zondervan-Zwijnenburg, Nancy E. van Loey

**Affiliations:** 1 Department of Methods and Statistics, Utrecht University, Utrecht, The Netherlands; 2 Optentia Research Program, Faculty of Humanities, North-West University, Vanderbijlpark, South Africa; 3 Department of Clinical & Health Psychology, Utrecht University, Utrecht, The Netherlands; 4 Department Behavioural Research, Association of Dutch Burn Centres, Beverwijk, The Netherlands

**Keywords:** Bayesian estimation, maximum likelihood, prior specification, power, repeated measures analyses, small samples, burn survivors, mechanical ventilation, PTSS

## Abstract

**Background:**

The analysis of small data sets in longitudinal studies can lead to power issues and often suffers from biased parameter values. These issues can be solved by using Bayesian estimation in conjunction with informative prior distributions. By means of a simulation study and an empirical example concerning posttraumatic stress symptoms (PTSS) following mechanical ventilation in burn survivors, we demonstrate the advantages and potential pitfalls of using Bayesian estimation.

**Methods:**

First, we show how to specify prior distributions and by means of a sensitivity analysis we demonstrate how to check the exact influence of the prior (mis-) specification. Thereafter, we show by means of a simulation the situations in which the Bayesian approach outperforms the default, maximum likelihood and approach. Finally, we re-analyze empirical data on burn survivors which provided preliminary evidence of an aversive influence of a period of mechanical ventilation on the course of PTSS following burns.

**Results:**

Not suprisingly, maximum likelihood estimation showed insufficient coverage as well as power with very small samples. Only when Bayesian analysis, in conjunction with informative priors, was used power increased to acceptable levels. As expected, we showed that the smaller the sample size the more the results rely on the prior specification.

**Conclusion:**

We show that two issues often encountered during analysis of small samples, power and biased parameters, can be solved by including prior information into Bayesian analysis. We argue that the use of informative priors should always be reported together with a sensitivity analysis.

“The bigger the better, in everything,” Freddie Mercury answered when he was asked if he was intimidated by a large audience (Wigg, 2012). A statement highly contrasting to the famous motto of Ludwig Mies van der Rohe: “less is more” (Mies van der Rohe, 1994). Although both quotes had nothing to do with statistics, they are representative for issues many researchers have to deal with. The quote of Freddie Mercury is highly applicable, because researchers in many research areas are expected to work with large sample sizes, especially in combination with complicated statistical models. As is widely understood, the bigger the sample size the higher the probability of finding a significant result (Peto et al., 1976). However, a large sample size is not always obtainable in any field of research. A small sample size can be the consequence of research protocols, a small research population, or when it is ethically and morally unjustified to gather a larger sample. When the sample is limited in size, it is, because of power issues, often hard to get meaningful results (Button et al., 2013; Lee & Song, 2004; Price, 2012; Scheines, Hoijtink, & Boomsma, 1999).

Hence, the question every researcher is faced with “How large should my sample be?” This question can only be answered in relation to the number of parameters included in a model (e.g., regression coefficients, intercepts, residual variances). Lee and Song (2004) concluded that when using maximum likelihood (ML) estimation for structural equation models, sample sizes yield accurate results when the ratio of parameters: sample size is 1:5, whereas a 1:3 ratio produced some bias. This ratio is less stringent than the rules of thumb often used in practice, for example, 1:10 or 1:20. Of notice, the ratio also depends on the model, the distribution of the parameters/variables and other characteristics. As argued by Muthén and Muthén (2002), the only way to answer the sample size question is by performing a simulation study. If a simulation study shows that a rule like 1:5 results in sufficient power, it would provide a starting point for practical purposes.

However, what if the sample size based on such a ratio is simply not feasible? Consider the hypothesis that burn survivors who need mechanical ventilation in response to inhalation injury have higher levels of posttraumatic stress symptoms (PTSS) over time relative to burn survivors who do not need mechanical ventilation. In such an example, obtaining a large sample is difficult in a timely fashion because of the relatively low incidence of burn injuries and particularly comorbid inhalation injury. Approximately, 10% of burn patients admitted to a burn center have inhalation injury (Belgian Outcome in Burn Injury Study Group, 2009). Moreover, only a minority of the patients develops PTSS (Van Loey & Van Son, 2003). According to a power study, 66 burn survivors would be required of which half require mechanical ventilation.^1^ To gather such a sample in small countries like The Netherlands or Belgium would take many years. It could be the case that, different underlying mechanisms may play a role in the development and maintenance of PTSS in those with and without ventilation. Moreover, different strategies may be required to treat or even prevent this disorder. However, the power analysis tells a researcher that in order to detect such a difference, a sample size is needed that is, without an unreasonable amount of time and resources, unattainable. Should a researcher then simply ignore this research interest/question? If the research is never carried out, then, a possible important risk factor may be overlooked resulting in inadequate treatment with long term consequences such as lower quality of life (Van Loey, Van Beeck, Faber, Van de Schoot, & Bremer, 2012). Shouldn't science also aim at answering research questions of great importance highly relevant for small research populations?

Because Bayesian analyses do not assume large samples, as is the case with ML estimation, typically smaller data sets can be analyzed without losing power while retaining precision. As Lee and Song (2004) showed, Bayesian estimation requires a much smaller ratio of parameters to observations, namely for the models used in their paper a ratio of 1:3 could be used instead of 1:5. Also Hox, Van de Schoot, and Matthijsse (2012) showed with a simulation study that using Bayesian estimation makes it possible to use smaller data sets compared to ML estimation (see also Hox, Moerbeek, Kluytmans, & Van de Schoot, 2014).

Because the aim of our study is not to introduce Bayesian statistics, we kindly refer readers who are interested to the gentle introduction of Van de Schoot et al. (2014), but see also Kaplan and Depaoli (2012), Kruschke, Aguinis, and Joo (2012), or Zyphur and Oswald (2015). For more technical details we refer to Gelman, Carlin, Stern, and Rubin (2004). The heart of Bayesian estimation is that everything that is known about a parameter before observing the data (the prior) is combined with the information from the data itself (the likelihood), resulting in updated knowledge about the parameter (the posterior). The prior information can stem from a meta-analysis, previous studies with comparable research populations, a pilot study, experts, or a range of other sources. If such knowledge is used we call the prior *informative*, and if no knowledge is available (or used) we call the prior *non-* or *un-informative*. It's debatable when a prior is considered informative or un-informative, therefore different statistical programs use different default settings regarding the un-informative priors. An often used un-informative prior for variance terms is an improper prior. An improper prior is a probability distribution that does not sum up or integrate to one (Jackman, 2009). Because it does not integrate or sum to one, it can technically not serve as a probability distribution. We return to this issue later in the article.

In the study of Lee and Song (2004) the authors considered a situation in which, *a priori*, there was no information available about the parameters of interest. As such, un-informative prior distributions were specified for the parameters in the model. Hence, the full potential of the Bayesian toolbox was not used. Galindo-Garre, Vermunt, and Bregsma (2004) specified an informative prior and the authors concluded that the more information is captured by the well-specified priors the smaller the parameter bias. Also, (Depaoli, 2012) compared ML estimation and the Bayesian framework, but she implemented a wide range of priors: diffuse/un-informative priors, “accurate” informative priors, weakly informative priors, data-driven informative priors, priors reflecting partial-knowledge of parameters, and “inaccurate” (but informative) priors. The results indicated that optimal parameter recovery was obtained through the Bayesian approach using “accurate” informative priors, and partial-knowledge priors showed promise for the recovery of the growth trajectory parameters. Price (2012) compared the small sample performance of a Bayesian versus a frequentist time series model on power and parameter estimation bias. Using informative priors, Price showed the Bayesian approach provided better results for hypothesis testing and detecting Type I error compared to ML. We extended the work of the authors discussed above in two ways. First, we investigate what influence the amount of information captured by prior distributions has on the ratios between the number of parameters in the model and required sample size. Second, we consider more extreme parameter to sample size rations. We expect Bayesian estimation to give reliable results with very small sample sizes *if* prior information is added to the analyses.

In sum, our main objective of this study is to demonstrate possibilities that exist for researching small populations through the use of Bayesian estimation, but as we show the use of Bayesian statistics comes with a price. That is, background information needs to be incorporated in the analyses via the prior distribution. We demonstrate (1) how to specify these priors; (2) how to investigate the exact influence of the prior specification on the results and conclusions; and (3) how much can be gained in terms of sample size if more information is specified via the priors. Throughout the paper a dataset concerning PTSS in burn survivors is used as a case study to demonstrate the advantages *and* pitfalls of using Bayesian statistics to deal with small research populations. In particular, it was investigated whether a period of unconsciousness during mechanical ventilation would be associated with a different course of PTSS. As the number of burn survivors is limited we have to deal with a small sample size.

The structure of this paper is as follows: First, PTSS in our sample is introduced and a description is given of Repeated Measures ANOVA (RMA). Then we present the results of the sensitivity analysis (Study 1) where we investigate the influence of the prior specification on the results. Thereafter, in a second study, we present a simulation where we investigate the relationship between the required sample size and the specification of the prior distribution. Finally, the data on burn survivors is analyzed. The data and syntax files for the sensitivity analysis and the simulation study can be found on the website of the first author.^2^

## Empirical example

### Posttraumatic stress disorder

Posttraumatic stress disorder (PTSD) is a mental health condition that can develop in the aftermath of exposure to a traumatic event. It is one of the most common psychiatric diagnoses in the general population after trauma exposure (Breslau et al., 1998). PTSD includes four symptoms clusters: intrusion, avoidance, negative alterations in cognitions and mood, and alterations in arousal and reactivity (American Psychiatric Association, 2013). A dysregulated fear response is considered an important mechanism underlying PTSD. Fear conditioning is a complicated process in which a neutral conditional stimulus becomes associated with an aversive unconditioned stimulus (Parsons & Ressler, 2013). After the fear conditioning the traumatic experience is consolidated in the memory. The consolidation period is a time span directly followed after the trauma exposure; this period could last minutes, hours, or even days (Parsons & Ressler, 2013). Memory consolidation is a process in which a transformation over time is going on from short-term memory into long-term memory (Schafe, Nader, Blair, & LeDoux, 2001). In recent years interest has grown in factors that affect the consolidation process (Baldi, Liuzzo, & Bucherelli, 2013; Datta & O'Malley, 2013; Zohar, Juven-Wetzler, Myers, & Fostick, 2008). However, there is a paucity of studies investigating whether a period of mechanical ventilation in which the patient is brought into a state of unconsciousness, would be associated with a different PTSD course over time. A possible underlying mechanism may be that the consolidation process during this period may be reinforced. A recent review reported that three out of four studies performed in ICU including a wide range of pathologies, identified mechanical ventilation to be a risk factor for PTSS (Wade, Hardy, Howell, & Mythen, 2013). Although the results are currently inconclusive, possibly, the period of ventilation may reinforce the consolidation process during this period in such a way that the fear conditioning transforms more easily to a more permanent state over time. As no studies are available in burn injured populations, the research question of interest is whether the development of PTSS in burn survivors who need mechanical ventilation is different compared to burn survivors who do not need mechanical ventilation.

### Method

A subsample of the data from Van Loey, Maas, Faber, and Taal (2003) was considered. Our subsample encompassed persons scoring above the cutoff point of clinically relevant symptom levels measured 2 weeks post burn, and for whom data on mechanical ventilation status was available. Data were gathered in burn centers in the Netherlands and Belgium between 1997 and 2003. Patients admitted to a burn center were invited to participate in a longitudinal study on PTSS. Patients with cognitive disabilities, insufficient Dutch proficiency, and a length of stay in hospital less than 72 hours were excluded. Patients were invited to take part within 2 weeks post burn by a local researcher and after providing written informed consent, they completed questionnaires. Follow-up questionnaires were sent to their home address. Those requiring mechanical ventilation were included into the study as soon as they gained consciousness and were able to comprehend questions. The results were described in Van Loey et al. (2003).

**Fig. 1:**
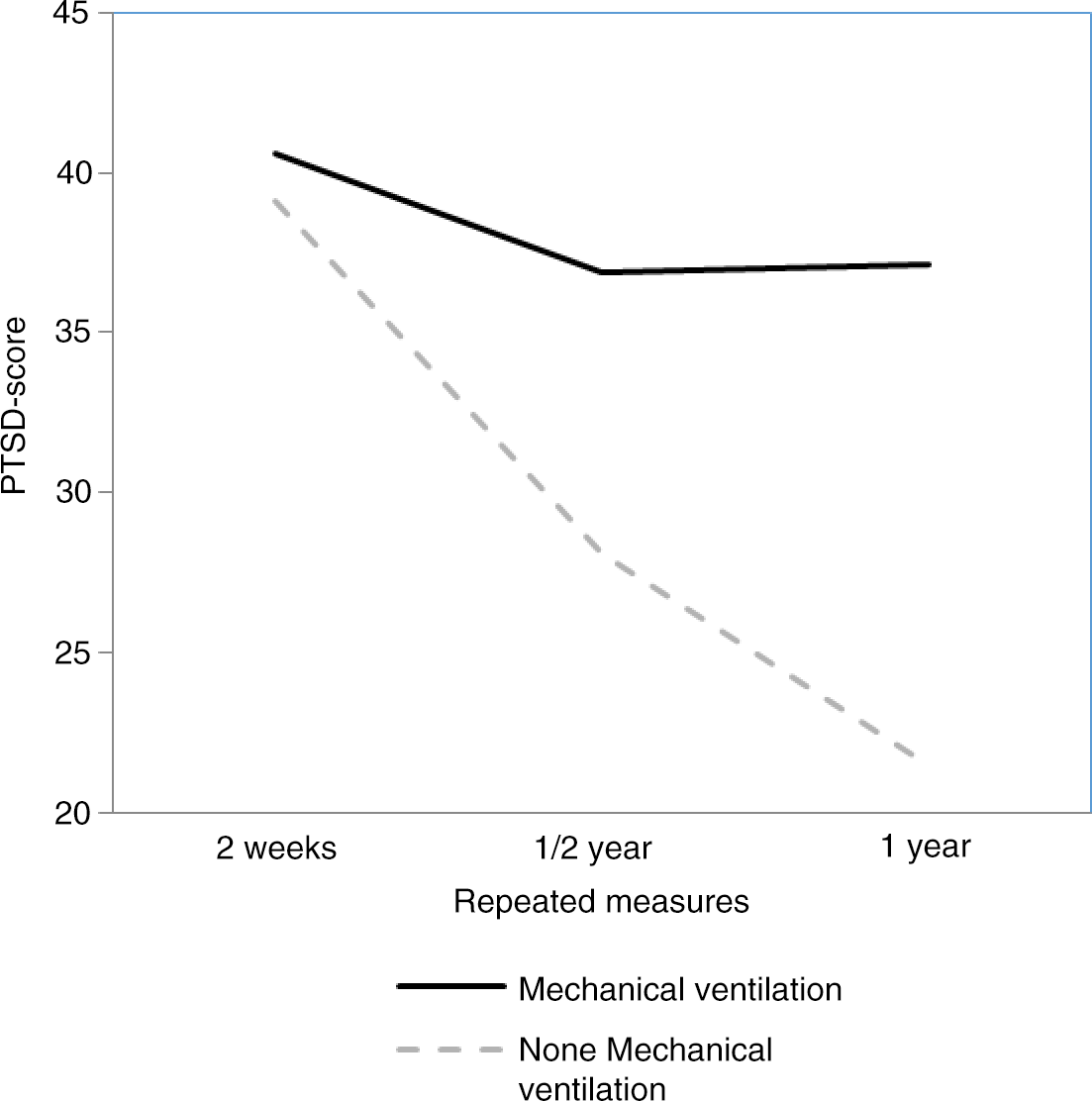
PTSS scores over time of mechanical ventilation and non-mechanical ventilation group.

PTSS were measured using the Impact of Event Scale (IES) (Horowitz, Wilner, & Alvarez, 1979). The IES is a 15-item self-report questionnaire used to assess intrusive and avoidant symptoms associated with the experience of a particular event. In this questionnaire, participants are requested to rate statements like “I thought about the burn event when I didn't mean to”, “I tried to remove the burn event from memory” and “I had trouble falling asleep or staying asleep because of pictures or thoughts about the burn event that came into my mind”. In this study, the Dutch version of the IES was used (Brom & Kleber, 1985). Responses were rated on a 0 (not at all) to 100 (the worst imaginable way) visual analogue scale. Measurements used in this example include 2-week, 6-month, and 12-month assessments. Figure 1 displays a graphical indication of the development of PTSS over time.

The study of Van Loey et al. (2003) featured a prospective longitudinal cohort design. All patients who gave written informed consent received questionnaires while they were admitted to the burn center. Once patients left the burn center, the staff member continued these follow-ups by regular mail, sending patients a stamped return envelope with the questionnaire. The original data set contains 301 participants. Our subsample of *n*=78 is based on complete cases of respondents who have a score of 25 or higher on IES 2 weeks after the burn event. As the IES cutoff point varies across studies between 19 and 35 (Wade, Hardy, Howell & Mythen, 2013), 25 was chosen as the point at which there is a considerable level of traumatic stress without unnecessarily further decreasing the patient group of interest. Although most burn events are potentially traumatic in nature, not all patients perceive the event as traumatic. For the purpose of this study, it was important to include patients with clinically relevant symptom levels to meet the theoretical assumption that trauma consolidation may be reinforced during a period of ventilation, a process that will not take place in patients scoring low on traumatic stress. In the original sample 24 participants scored 25 or higher after 2 weeks, but had missing values on the next two measures and so these participants were excluded from the analyses. However, compared to the included participants the participants with missing data did not significantly differ in length of hospital stay, age, total burned surface area, sex, and mechanical ventilation requirements (all *p*'s >0.42). Note that because of the small sample these analyses are low in power.

In the subsample 49 (62.8%) patients were male and 29 (37.2%) were female with an age ranging from 16 to 79 years (*M*=37.35, *SD*=12.73). The average length of hospitalization was 27.51 days (*SD*=30.22). The patients’ body surface area burned ranged from 1 to 60% (*M*=15.99%, *SD*=15.24%). Of the 78 patients, 15 (19.2%) persons needed mechanical ventilation and 63 (62.8%) did not need mechanical ventilation.

### Statistical analysis

To analyze the data, we used a RMA which is an often used method to analyze data on the same subjects over different time periods or under different experimental conditions. The design is used to test whether there are significant within or between subject differences over time or across conditions. Inspecting Fig. 1, the IES-scores seem to decrease earlier for subjects who did not require mechanical ventilation. According to a power study, 66 burn survivors would be required of which half require mechanical ventilation. For more details and the syntax files, see the website of the first author.^3^ As discussed in the Introduction section, it is very difficult to gather such a large sample. The main question we aim to answer in the current paper is whether Bayesian statistics can be used to overcome the limited data issue.

In the current study, we mimicked the RMA as obtained in SPSS version 20.0 (IBM Corp., 2011) using the software Mplus version 7.1 (Muthén & Muthén, 1998–2012) in order to vary estimators and to be able to perform the sensitivity analysis and the simulation study. These authors show that the RMA model can be expressed by a structural equation model which looks similar to a latent growth model, but with some extra restrictions. Although RMA is often used, it is not considered the best model to estimate longitudinal data (Davis, 2003), but with small data sets the number of parameters needs to be as small as possible. To do so, we applied the method as is discussed in Duncan, Duncan, and Strycker (2009, p. 42).

**Fig. 2:**
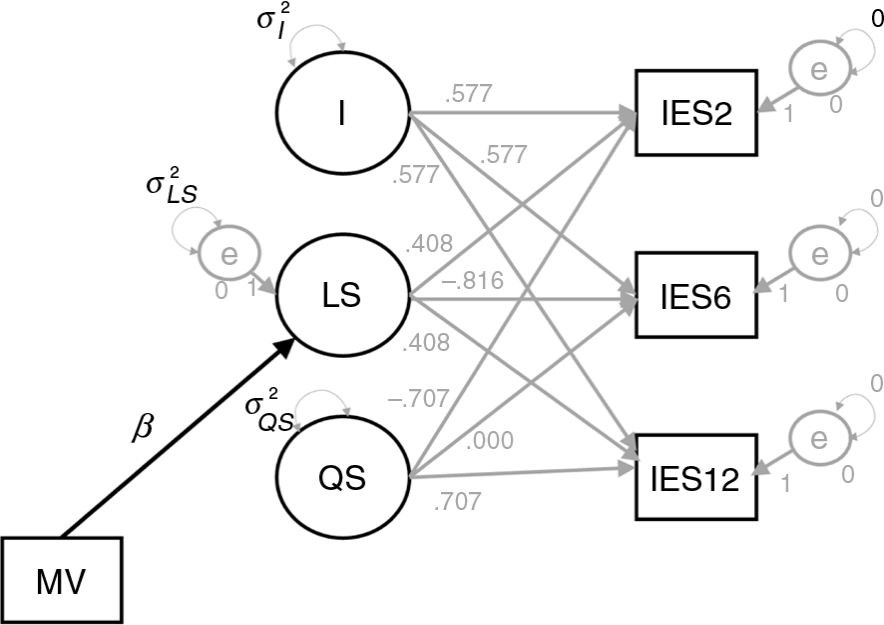
Estimated model in Mplus.

As shown in Fig. 2, the observed IES-scores are used to estimate the latent mean and variance for the Intercept (denoted by *I* and $\sigma_{I}^{2}$, respectively), the Linear Slope (LS and $\sigma_{\text{LS}}^{2}$), and Quadratic Slope (QS and $\sigma_{\text{QS}}^{2}$). Note that we fixed the covariances to zero to simplify the model even further. The observed variables are IES2 which contains the scores after 2 weeks, IES6 which contains the score after 6 months, and IES12 which contains the scores after 12 months. The factor loadings of *I*, LS, and QS are fixed, based on the transformation matrix derived from a three time-point RMA in SPSS using the method of Duncan et al. (2009, p. 42). The group effect is estimated by regressing LS on a dichotomous variable that indicates whether or not the burn survivors have had mechanical ventilation. Thus, the parameter of our main interest is indicated by *β*. The syntax to mimic RMA in Mplus can be found on the website of the first author.^4^

## Study 1: sensitivity analysis

### Specifying the prior distribution

In the present study, the prior distribution should reflect what we know about the parameters in the model shown in Fig. 2. The main parameter of interest is the difference in the slope between our two groups (denoted by *β*), but also, all the other parameters in the model of Fig. 2 need to have a prior distribution. In this study we differentiate between the structural parameters of the model (the intercept, the slope, the quadratic term, and *β*) and the (residual) variance terms (the variance of the intercept and the quadratic term, and the residual variance of the slope).

The specification of the prior distributions consists of three steps. First, background knowledge is needed as input for the specification. Such knowledge can stem from a meta-analysis, previous studies with comparable research populations, a pilot study, experts, or a range of other sources. In our case we used the systematic review of Wade et al. (2013) as inspiration. In the section *Results Empirical Example* we elaborate on this.

Second, for all parameters a type of distribution has to be specified. For the structural parameters we used a Normal distribution (denoted by N) and for the variance parameters we specified an Inverse Gamma distribution (denoted by IG).^5^

The third step of the prior specification is to determine the shape of the prior distribution by means of choosing values of the hyperparameters. In the case of the structural parameters, the hyperparameters are the mean of the normal distribution, denoted by *µ*_0_, and the variance of the prior distribution, denoted by $\sigma_{\text{0}}^{2}$. Note that we use subscripts 0 and 1 to denote whether we refer to the prior parameters (*µ*_0_ and $\sigma_{\text{0}}^{2}$), or posterior parameters (*µ*_1_ and $\sigma_{\text{1}}^{2}$). The prior mean expresses the expectation of the researcher with respect to the value parameter estimate and the prior precision or its inverse, the prior variance, expresses the researcher's certainty about the parameter value. For instance, a prior distribution with a prior variance of 1,000 expresses much more insecurity about the prior mean than a distribution with a prior variance of 1. For the variance parameters the hyperparameters are given by the scale (*α*_0_) of the distribution and the shape of the distribution (*υ*_0_). For more technical details of the implementation of the IG distribution in Mplus, see Asparouhov and Muthén (2010). For example, an IG distribution with hyperparameters *α*_0_=−1 and *υ*_0_=0 reflects a flat but positive distribution and *α*_0_=0.5 and *υ*_0_=0.5 reflects an informative distribution with values close to zero much more plausible.

In what follows, we discuss in great detail how we specified the specific hyperparameters for our PTSS-example and we investigate by means of a sensitivity analysis what influence the hyperparameters have on the results.

### Default prior settings

As discussed in the previous section, Bayesian estimation requires prior distributions for every parameter. First, the default settings of Mplus were used for the prior distributions (Asparouhov & Muthén, 2010). The default settings for the structural parameters are normal distributions with a prior mean of zero (*µ*_0_=0) and a very large prior variance resulting in an almost flat prior distribution (i.e., $\sigma_{0}^{2}$=10^10^). These priors indicate not much is known about the structural parameters of the model. So, if no background knowledge would be available, the default settings of Mplus could be used. For the variance parameters by default an Inverse Gamma distribution is used in Mplus with *α*_0_=−1 and *υ*_0_=0. These settings result in a flat but positive distribution, thereby making negative (residual) variances impossible, but no information is included about the shape of the distribution.

A fixed number of 10,000 iterations was used, and to check convergence we increased the number of iterations to 50,000 and 100,000 iterations. There appeared to be hardly any difference for *β* (max −0.33 and −0.80%, respectively). To decrease computational time we used 10,000 iterations for the remainder of our study.

The results of the analyses using ML and Bayesian estimation relying on the default prior settings are shown in Table 1. Only some very small numerical differences were obtained between the coefficients of ML and Bayesian estimation with default prior settings, but we consider these differences as ignorable.

**Table 1:** Coefficients obtained in Mplus using ML and Bayesian estimation with default prior settings

Type of analysis	*I*	S.E.	LS	S.E.	*β*	S.E.
ML	54.090	2.759	−12.481	1.779	10.008	4.057
Bayes	54.068	2.839*	−12.466	1.862*	9.940	4.228*

*Note*: *I*=Intercept; LS=Linear slope; *β*=difference between two groups; S.E.=standard error; ML=maximum likelihood estimation; Bayes=Bayesian estimation.

* Posterior S.D. instead of SE.

### Informative priors

In order to make use of the advantages of the Bayesian toolbox, the prior distributions have to be more informative than the default settings of Mplus. To investigate the influence of the prior specifications on the posterior results we performed a sensitivity analysis. First, we used a well-specified prior mean but varied the prior variance. Next, we varied the prior mean *and* the prior variance. Note that the priors should be based on background knowledge. In the section *Results Empirical Example* we actually use background information to specify the prior distributions. For the current section we aim to investigate how prior settings influence the results and therefore we examine a wide range of different prior specifications.

### Sensitivity analysis

#### Different values for the prior variance

In the first sensitivity analyses, a well-specified prior mean was used for *β* (*µ*_0_=10). That means that the prior mean is similar as for the ML-output. In this way, the *a priori* specified means are completely compatible with the data. Note that in Bayesian analysis, priors are by definition specified independent of the data to be analyzed. The goal of this part of our study is to show the performance of Bayesian analyses when only the prior variance is varied. In the next section, we investigate misspecification of the prior mean.

To investigate the influence of the prior variance on the posterior results we ran a sensitivity analysis with several values for the prior variance of *β*. Default prior settings were used for the other parameters in the model. We started with the ML results followed by the default prior settings ($\sigma_{0}^{2}$=10^10^). Then, we decreased the prior variance in several steps. A prior variance of $\sigma_{0}^{2}$=100 can still be considered non-informative, but a prior variance of $\sigma_{0}^{2}$=0.1 might be considered highly informative, maybe even too informative. However, see Van de Schoot et al. (2013) where such highly informative priors were used in the context of testing for measurement invariance. In our study, however, this is not the case.

**Fig. 3:**
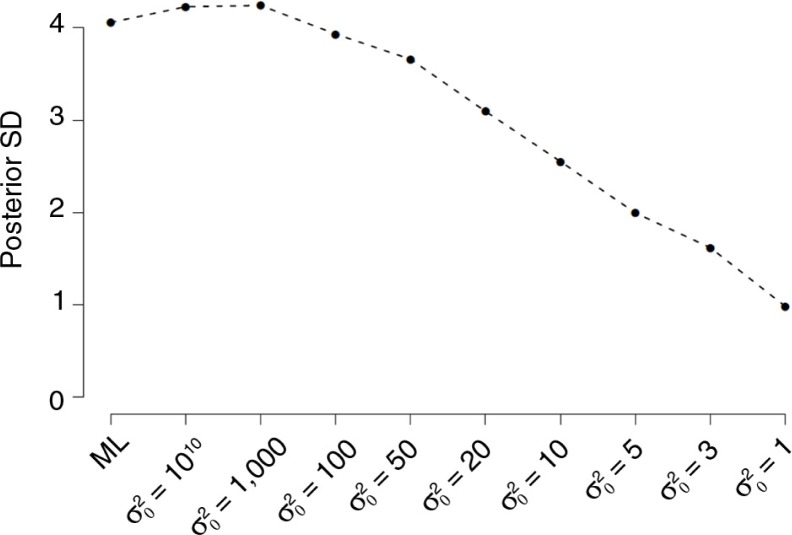
The effect of different estimators (ML vs. Bayes) and different values for $\sigma_{0}^{2}$, but with a fixed *µ*_0_ for *β*.

In Fig. 3 (see also Supplementary Table 1) the effect on the posterior standard deviation for *β* is displayed. As is clearly visible the smaller the specified prior variance, the smaller the posterior *SD* becomes. This means that the more information available in the prior the more precise the posterior results become.

#### Different values for the prior mean and prior variance

In order to study the influence of potential misspecification of the prior mean we varied both hyperparameters for *β*. According to the ML estimation *β* was estimated as 10, instead we used a prior mean of *µ*_0_=5, 0, −5, and −10 and at the same time we varied $\sigma_{0}^{2}$. The reason behind the values for =*µ*_0_ is that, based on background knowledge, we expected a difference in the opposite direction; see also the section *Results Empirical Example*.

**Fig. 4:**
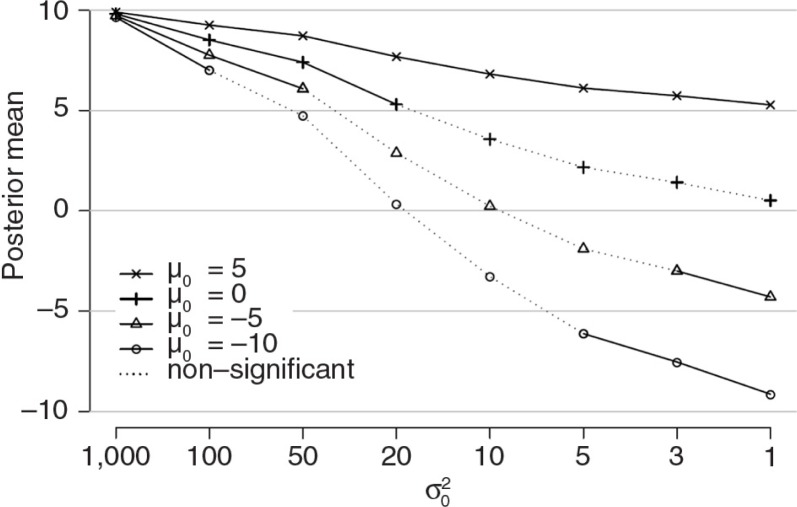
Influence of miss-specification of prior mean for *β* (*µ*_0_) on the posterior mean for *β* (*µ*_1_) with different prior variances ($\sigma_{0}^{2}$).

In Fig. 4 the results of the sensitivity analysis are displayed; see also Supplementary Table 2 for the numerical results. On the y-axis the posterior estimate for *β* is displayed, indicated by *µ*_1_ and on the x-axis the different values for the prior variance are displayed. The four lines represent the results for the four different prior means. We indicated whether the posterior estimate was non-significant^6^ by the dashed line.

The results show that no matter which prior mean we specified, using a prior variance of $\sigma_{0}^{2}$=1,000 the difference between the ML estimate of *β* (i.e., *β*=10) and the Bayesian estimate is non-existent. However, there was a difference between the ML result and the Bayesian result when the prior variance decreased. It appeared that when *µ*_0_=5 for all values of $\sigma_{0}^{2}$ the estimate of *β* was always significant. However, with *µ*_0_=0 depending on the specification for $\sigma_{0}^{2}$ the estimate of *β* was either significant ($\sigma_{0}^{2}$=20–1,000) or not significant ($\sigma_{0}^{2}$=1–10). For *µ*_0_=−5 and *µ*_0_=−10 the influence on the conclusion is even more substantial. That is for some values for $\sigma_{0}^{2}$ the estimate for *β* is significant for an effect in the opposite direction to that found via ML (see the solid lines in the bottom-right corner of Fig. 4).

In conclusion, the smaller the prior variance for *β*, the more influence the prior specification of *µ*_0_ has on the posterior results, and in some cases the conclusions might even change.

### Intermediate conclusion

When specifying subjective priors the conclusions might be affected by the choice of the hyperparameters for the prior distributions. A researcher should always make strong arguments why he or she has chosen the specific hyperparameters and we recommend presenting the results of a sensitivity analyses, as in Fig. 4, to investigate the robustness of the conclusion with respect to the prior specification. But we urge researchers not to change the priors if such a prior-data conflict is encountered, and rather spend a paragraph in the discussion section explaining this conflict.

## Study 2: simulation study

### Simulation design

A simulation study was conducted to examine the performance of ML and Bayesian estimation, with different prior settings, and to determine the required sample size to obtain enough power. The statistical model in the simulation design was identical to the model we used for the sensitivity analysis (Fig. 2). This model contains seven parameters: the mean and variance of the intercept, the mean and residual variance for the slope, the mean and variance for the quadratic trend, and *β*. All population values for the simulation study were based on the ML results using the PTSS data. Thousand datasets were created with sample sizes of *n=*8, 14, and 22.

The datasets were analyzed using ML, and Bayesian estimation with different prior settings. In the Bayesian analyses the hyperparameters were specified for *β* (*µ*_0_=10; $\sigma_{0}^{2}$=10^10^, 1,000, 100, 50, 20, 10, 5, 3, 1). As we discuss below, we encountered an issue with the prior distribution for the variance terms, therefore we ran an additional simulation study where we also varied the hyperparameters for the IG distribution. All analyses were run with 1,000 replicated datasets and 10,000 iterations for each data set. We focused on four outcome criteria:

1. The relative mean bias defined as $((\bar{\theta }-\theta )/\theta )\cdot 100$, where $\bar{\theta }$ is the average mean obtained from the simulation study, and θ is the population value. We used a cutoff value of <10% in absolute value as a criterion, as suggested by Hoogland and Boomsma (1998) for “reasonable” accuracy.
2. The 95% coverage of the population value across replications.
3. The percentage of significance results across replication of the specific parameter as an indication of the power.
4. The mean square error (MSE).

### Simulation results

#### Results for maximum likelihood

In Table 2, the results of the simulation study with ML estimation is shown for a sample of *n=*8. Several conclusions can be drawn from these results. First, the bias in the LS and the QS variance exceeds 10%. Second, the coverage of all the parameters does not remain between the preferred values of 90–98% as specified by Muthén and Muthén (2002). The parameter of interest, *β*, is found to be significant in only 28.3% of the datasets (see the column labelled “power”).

**Table 2:** Results of simulation study with ML estimation

	*n*=8					
	
	Pop	Mean	Bias (%)	95% coverage	Power	MSE
*I*	54.0900	54.1551	0.12	0.907	1.000	74.0206
LS	−12.4810	−12.5464	0.52	0.853	0.557	51.6079
QS	1.7590	1.6870	4.09	0.880	0.137	17.2733
*β*	10.0080	9.9196	−0.88	0.864	0.283	100.8752
$\sigma_{I}^{2}$	593.6330	542.7769	−8.57	0.800	1.000	78909.3359
$\sigma_{\text{LS}}^{2}$	199.3820	152.9436	−23.29	0.695	1.000	9769.9697
$\sigma_{\text{QS}}^{2}$	134.6140	114.1505	−15.20	0.756	1.000	4169.3696

*Note*: *I*=Intercept; LS=Linear Slope; *β*=difference between two groups; *σ* ^2^=variance; POP=population value; MSE=mean.

#### Results for Bayesian estimation with default prior settings

In Table 3, the results of Bayesian simulation study with default prior settings is shown. Bayesian estimation shows some notable bias, in particular on the variances. For example, $\sigma_{\text{LS}}^{2}$ is estimated with a bias of 84.42% and the estimate for the variance of the intercept is not even provided by Mplus because it is too large. So, before we continue with the remaining cells of the simulation design, the issue of the large bias in estimating the variances in Bayesian estimation needs elaboration. The issue may be caused by some of the default settings used by Mplus. According to Asparouhov and Muthén (2010) the prior for (residual) variances can be important for small sample sizes. Therefore, we repeated the simulation study with different specifications for the inverse gamma distribution, IG(α_0_, v_0_).

**Table 3:** Results of simulation study with Bayesian estimation with default prior settings

	*n*=8					
	
	Pop	Mean	Bias (%)	95% coverage	Power	MSE
*I*	54.0900	53.8515	−0.44	0.981	0.993	74.0908
LS	−12.4810	−12.2739	−1.66	0.982	0.161	51.6691
QS	1.7590	1.8140	3.13	0.972	0.033	17.2806
*β*	10.0080	9.9278	−0.80	0.987	0.049	100.8716
$\sigma_{I}^{2}$	593.6330	1004.8042	69.26	0.918	1.000	********
$\sigma_{\text{LS}}^{2}$	199.3820	367.7034	84.42	0.906	1.000	72300.7656
$\sigma_{\text{QS}}^{2}$	134.6140	208.4106	54.82	0.923	1.000	17948.2188

******** =Mplus did not provide output due to a too large estimate.

*Note*: *I*=Intercept; LS=Linear Slope; *β*=difference between two groups; *σ* ^2^=variance; POP=population value; MSE=mean.

#### Results for different prior settings of the IG distribution

**Fig. 5:**
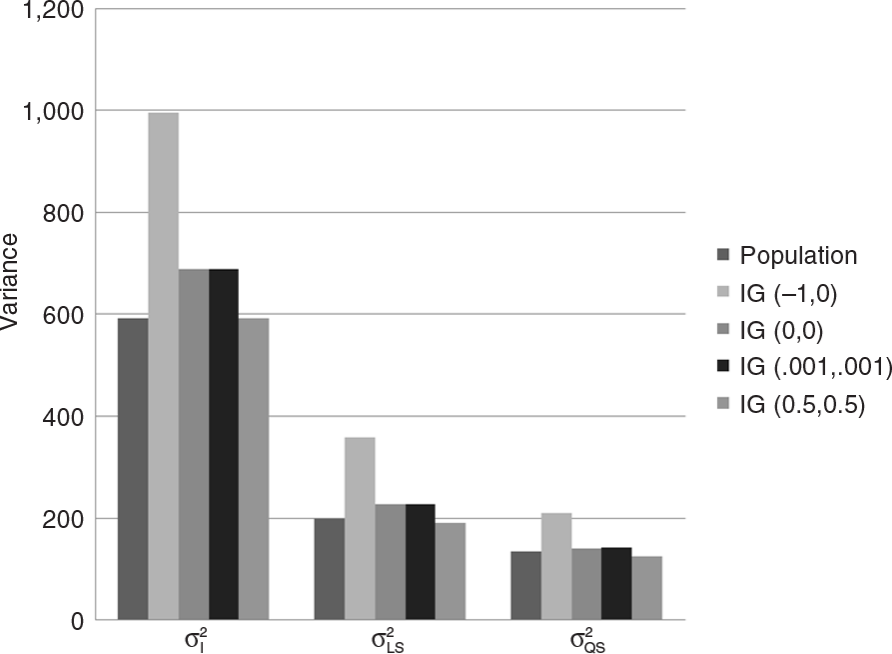
Simulation results for different priors for IG(α_0_, v_0_).

Additionally to the default setting for the priors for the variance parameters, IG(−1,0), we used three other specifications: IG(0,0), IG(0.001,0.001), and IG(0.5,0.5). In Fig. 5 (for numerical results, see Supplementary Table 3), the average across the 1,000 datasets is shown for the three variance terms and the four different specifications of the IG distribution. As can be seen in this figure, only the specification with IG(0.5,0.5) comes close to the population parameters. Let us elaborate what goes amiss.

**Fig. 6:**
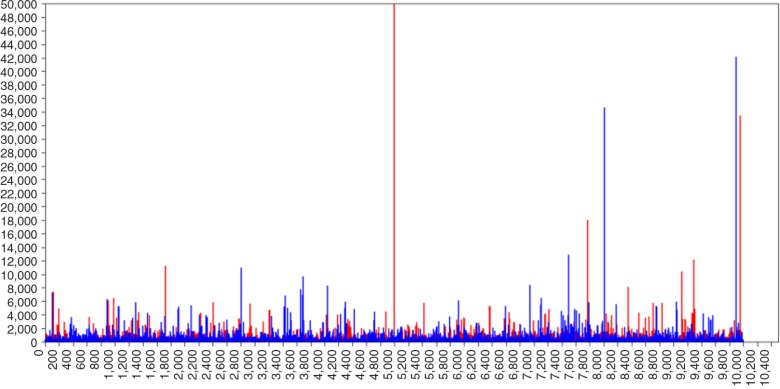
Trace plot of $\sigma_{\mathrm{LS}}^{2}$ with IG(−1,0)

**Fig. 7:**
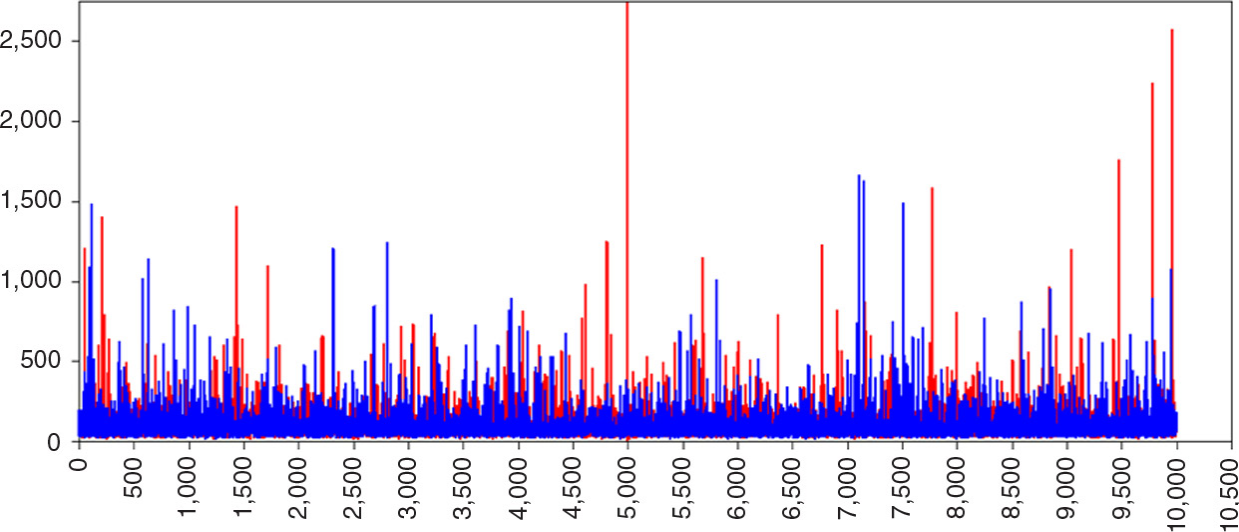
Trace plot of $\sigma_{\mathrm{LS}}^{2}$ with IG(0.001,0.001).

**Fig. 8:**
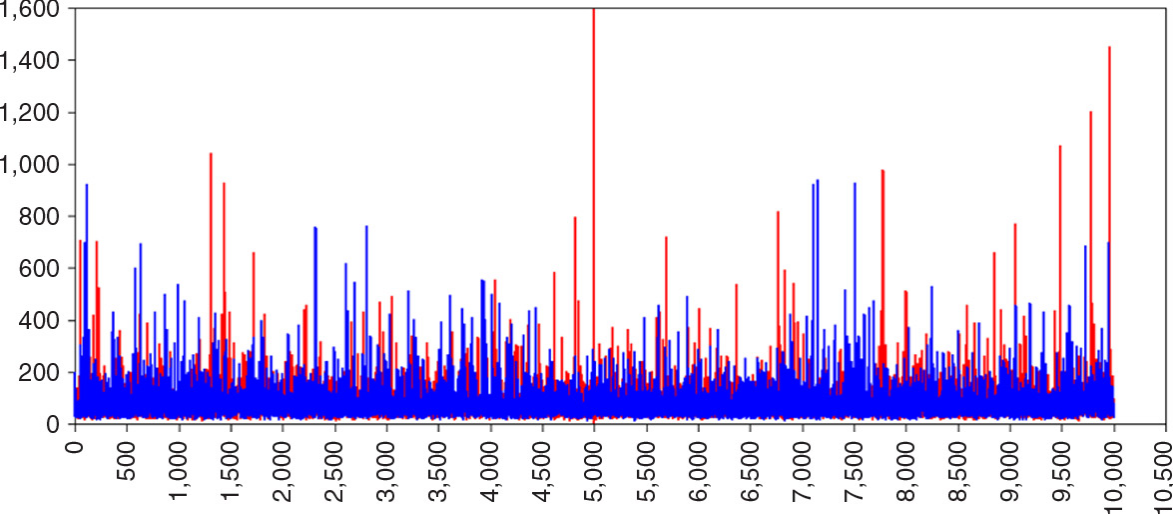
Trace plot of $\sigma_{\mathrm{LS}}^{2}$ with IG(0.5, 0.5).

The biased parameter results for the (residual) variance parameters can be explained by inspecting the trace-plot of, for example, the variance of the slope parameter of a randomly chosen dataset (i.e., #23). In Fig. 6, the trace plot of the variance of slope parameter with default prior settings is shown. Remarkably, this plot shows many large and extreme spikes with estimates of over 60,000 for $\sigma_{\mathrm{LS}}^{2}$. These extreme estimates are the source of the overestimation of the variance terms in Table 2. When specifying IG(0,0) we encountered the same issue. When using the prior specifications IG(0.001,0.001) the amount of unusual spikes decreased and peak values are less extreme. In Fig. 7, the trace plot is shown, but although these settings are recommended by Spiegelhalter, Thomas, Best, Gilks, and Lunn (1994, 2003), the trace plot still shows spikes. Only with a very informative IG distribution, with hyperparameters (0.5,0.5) the spikes disappeared (Fig. 8) and the posterior parameter value is closest to the population value, (Fig. 5).

An explanation is that the default hyperparameters for IG(−1,0) result in priors that are improper. An Inverse Gamma probability distribution is proper, when the shape and scale parameter are larger than zero, which is not the case for the two IG distributions with hyperparameters (−1,0) and (0,0), both shape and scale parameter are either equal or smaller than zero. This can cause computational problems (Asparouhov & Muthén, 2010). Therefore, it is recommended to always use proper prior distributions, for example, the prior distributions with hyperparameters (0.001,0.001) or (0.5,0.5).

The Bayesian simulation study shown in Table 3 was re-estimated with the IG(0.5,0.5) prior. As shown in Table 4, the percentage of bias of all the parameters in the model now remains under 10%. The 95% coverage of all the parameters stays between 0.91 and 0.98 and the power of the parameter of interest (*β*) increased from 0.049 (Table 3) to 0.168 (Table 4).

**Table 4:** Results of simulations study with Bayesian estimation with *IG*(0.5,0.5)

	*n*=8					
	
	Pop	Mean	Bias (%)	95% coverage	Power	MSE	MSE
*I*	54.0900	53.9523	−0.25	0.940	1.000	74.0450
LS	−12.4810	−12.4721	−0.07	0.928	0.408	51.6095
QS	1.7590	1.7501	−0.51	0.927	0.086	17.2724
*β*	10.0080	9.8491	−1.59	0.939	0.168	100.8948
$\sigma_{I}^{2}$	593.6330	593.6693	0.01	0.933	1.000	91253.5078
$\sigma_{\text{LS}}^{2}$	199.3820	192.3111	−3.55	0.940	1.000	12071.8916
$\sigma_{\text{QS}}^{2}$	134.6140	121.8294	−9.50	0.935	1.000	4424.4517

*Note*: *I*=Intercept; LS=Linear Slope; *β*=difference between two groups; *σ* ^2^=variance; POP=population value; MSE=mean.

#### Results for informative prior settings for β

**Fig. 9:**
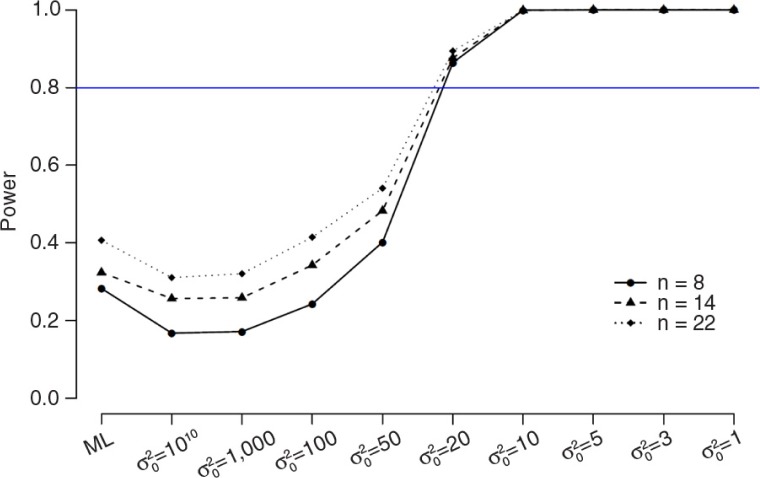
The results of the simulation study in terms of power.

**Fig. 10:**
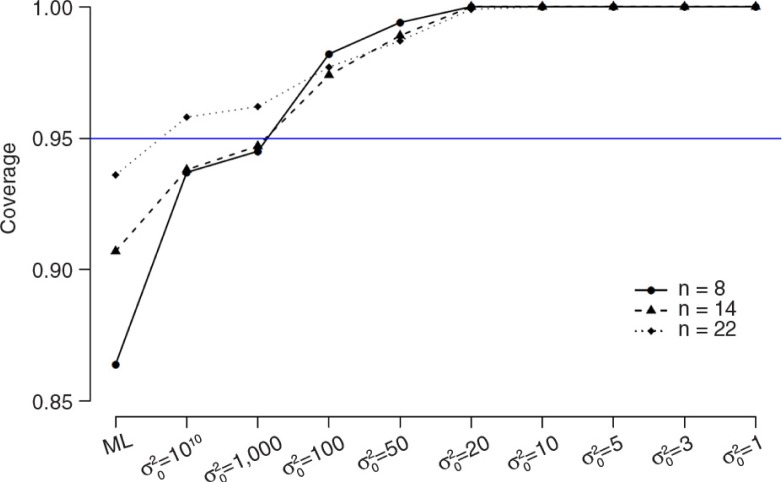
The results of the simulation study in terms of coverage.

As was demonstrated above, the bias of the parameters is well within acceptable limits, but the power of *β* is still dramatically low. Therefore, we specified different values for the prior variance for *β* ($\sigma_{0}^{2}$=10^10^, 1,000, 100, 50, 20, 10, 5, 3, 1) for *n*=8, 14, 22, respectively. In Figs. 9 and 10 (for numerical results, see also Supplementary Tables 4–6) the results for *β* of the simulation study with *n=*22 (Supplementary Table 4), *n=*14 (Supplementary Table 5), and *n=*8 (Supplementary Table 6) are displayed in terms of power (Fig. 9) and coverage (Fig. 10). Power is indicated by the proportion of simulations for which the estimate of *β* was significant. Coverage is indicated by the proportion of simulations which the estimate of *β* falls within the confidence interval.

As can be seen in both figures, the power and coverage of *β* increases with Bayesian estimation compared to ML estimation and also increased when the prior variance becomes lower. This effect is similar across the three sample sizes.

### Intermediate conclusion

In conclusion, the estimation of the posterior variance coefficients in small samples is highly dependent on prior specifications of the (residual) variance parameters. The default settings of Mplus might not be appropriate. Our recommendation is to always critically inspect the (residual) variance results and change the default prior settings if extreme values are obtained. With regard to the simulation results, as expected, the power of *β* increases in Bayesian estimation when the prior variance becomes lower and this effect is similar across the three sample sizes.

## Results empirical example

Because our study is the first to compare the development of PTSS in burn survivors who need mechanical ventilation and burn survivors who do not need mechanical ventilation, we used the following prior specifications:

- For the variance terms we specified an inverse gamma distribution with the hyperparameters IG(0.5,0.5) because these values appeared to be best according to our simulation study.
- For *β*, the regression coefficient denoting the difference in the slope parameter between the two groups, we specified a normal distribution with a prior mean of 0 and a variance of 20. The prior mean is based on our background knowledge that there are no previous studies conducted in this patient population and that studies with ICU population show conflicting/inconclusive results. That is, some suggest a negative effect, whereas other studies suggest no effect. The prior variance is chosen based on the result of our simulation study and because with a prior variance of 50, the 95% Posterior Probability Interval (PPI) of the prior distribution contains all plausible values for *β* (95% PPI=−14–13) and can be considered weakly informative.
- For the other parameters we relied on the default settings of Mplus.

We ran the Bayesian model with 10,000 iterations and inspected all trace plots for anomalies; there were none. The Mplus output file can be found on the website of the first author.^7^

The Bayesian results indicate that there is a significant group difference in the development of PTSS over time (*β=*7.456; *p*=0.02; 95% PPI=0.380–14.533). The posterior estimate is slightly pulled towards our prior mean as became apparent from our sensitivity analyses (Fig. 4). Thus, the effect in the data is stronger than we expected before analyzing the data. Because this is the first attempt to investigate an effect of mechanical ventilation in patients with burns, more research in the specific population of burn victims is needed to determine whether the effect can be replicated.

## Conclusion

This article demonstrated the influence of estimation techniques in RMA when dealing with small samples. In general, Bayesian analyses showed higher coverage than default ML estimation. When analyses with sample sizes of one time and two times the amount of parameters are conducted with ML, the coverage is insufficient, as was also concluded by Lee and Song (2004). In our study, both ML and Bayesian estimation show rather low power with small samples. Bayesian analyses with informative priors lead to more reliable results in terms of parameter bias and an increase of power, even when the sample size is equal to the amount of parameters. The robustness of results, however, highly depends on specification of the hyperparameters as we showed with our sensitivity analysis. This does not only concern the hyperparameters of the parameter of interest but also the hyperparameters of the (residual) variance terms. As shown, the default prior settings of variance terms in Mplus cannot always be trusted with small samples.

We also acknowledge some limitations of our study. The first is the use of the repeated measure model which is often used but also generally considered not to be the most appropriate procedure for analysis of repeated measures data, see e.g., Davis (2003). Better methods would be latent growth modeling or mixed effects modeling. Such models have more flexibility, but at the same time also have more parameters. Our main conclusions would not change for the alternative model, that is, the smaller the sample size, the more issues with ML estimation. Bayesian priors can be helpful in these situations, but this method is not without dangers itself and careful examination of the results is needed as well as a sensitivity analysis.

Another limitation is the way we treated missing data. In particular, data from individuals with incomplete data have been discarded. In the present data set this is not a strong limitation because these individuals only have baseline values recorded, but this is often not the case in other longitudinal data. We refer to Asendorpf, Van de Schoot, Denissen, and Hutteman (2014) for ways how to deal with longitudinal missing data.

Also note that the use of *p*-values, either frequentist or Bayesian, is not without issues. There is a well-known shift from this concentration on *p*-values, either frequentist or Bayesian, toward more emphasis on estimated relationships, confidence (or credibility) intervals, clinical relevance, and replication (see Asendorpf et al., 2013). A final remark concerns the dependence on prior specifications in Bayesian estimation, which, might be seen as controversial, because of their subjective nature. It requires expert knowledge about the use of prior specifications and the research topic. Statistical analyses are usually connected with objectivity, but Bayesian estimation brings in a more subjective element. If and how prior knowledge can be incorporated raises philosophical issues. Nevertheless, a small research population, like burn survivors but also multicenter studies, could benefit from the techniques described in this paper.

From a practical point of view, we advise ML or objective Bayesian estimation when the sample size is equal to or larger than three times the amount of parameters. Although Bayesian estimation provides reliable results when sample sizes are small, power can remain an issue. When prior information is available, subjective priors can offer the opportunity for research on small research populations to increase accuracy, coverage, and power. But such analyses should always be accompanied by a sensitivity analyses to investigate the influence of the specification of the hyperparameters of the prior distribution.

## Supplementary Material

Analyzing small data sets using Bayesian estimation: the case of posttraumatic stress symptoms following mechanical ventilation in burn survivors

Analyzing small data sets using Bayesian estimation: the case of posttraumatic stress symptoms following mechanical ventilation in burn survivors

## Footnotes

1 We estimated the power using a repeated measure model with three outcome variables and regressing a dichotomous variable on the slope and parameters based on the empirical data. For more information and the syntax files go to the website of the first author.

## Footnotes

2 http://rensvandeschoot.com/433/additional-information-analyzing-small-data-sets-using-bayesian-estimation.

## Footnotes

3 http://rensvandeschoot.com/433/additional-information-analyzing-small-data-sets-using-bayesian-estimation.

## Footnotes

4 http://rensvandeschoot.com/433/additional-information-analyzing-small-data-sets-using-bayesian-estimation.

## Footnotes

5 Note that because we constrained the covariances between the latent variable to zero, an IG distribution is used in Mplus instead of the multivariate Inverse Wishart distribution, see Asparouhov and Muthén (2010).

## Footnotes

6 “The Bayesian counterpart of the frequentist confidence interval is the Posterior Probability Interval (PPI), also referred to as the credibility interval. The PPI is the 95% probability that in the population the parameter lies between the two values. Note, however, that the PPI and the confidence interval may numerically be similar and might serve related inferential goals, but they are not mathematical equivalent and conceptually quite different” (Van de Schoot et al., 2014, p. 849).

## Footnotes

7 http://rensvandeschoot.com/433/additional-information-analyzing-small-data-sets-using-bayesian-estimation.
