# Supplementary material for: Analyzing small data sets using Bayesian estimation: the case of posttraumatic stress symptoms following mechanical ventilation in burn survivors
Source: Eur J Psychotraumatol. 2015 Mar 11;6:10.3402/ejpt.v6.25216. doi: 10.3402/ejpt.v6.25216 (PMC4357639; doi:10.3402/ejpt.v6.25216)
Supplement: Analyzing small data sets using Bayesian estimation: the case of posttraumatic stress symptoms following mechanical ventilation in burn survivors [file EJPT-6-25216-s001.pdf]

Rens van de Schoot, Joris J Broere, Koen H. Perryck, Mariëlle Zondervan-Zwijnenburg, Nancy E.E. van Loey

Abstract
Analiza małych zbiorów danych przy użyciu estymacji bayesowskiej oraz efektu uprzedniej specyfikacji: wpływ mechanicznej wentylacji po oparzeniach na przebieg objawów potraumatycznych.

Analiza małych zbiorów danych (ang. the analysis of small data sets) w badaniach podłużnych może wiązać się z problemami metodologicznymi dwojakiego rodzaju: problemem mocy wnioskowania oraz problemem niedokładności oszacowania parametrów. Kwestie te zostały zademonstrowane w badaniach dotyczących wpływu mechanicznej wentylacji po oparzeniach na przebieg objawów potraumatycznych. Wykazaliśmy, że powyższe problemy mogą zostać rozwiązane poprzez wprowadzenie wcześniejszych danych w analizie bayesowskiej. Jeśli badacz chce użyć estymacji bayesowskiej, musi dokonać uprzedniej specyfikacji rozkładu danych (ang. prior specification of distributions). W naszych badaniach okazało się, że im mniejsza wielkość próby, tym bardziej wyniki zależą od procesu uprzedniej specyfikacji. Jednakże dokonując estymacji metodą największego prawdopodobieństwa wykazaliśmy również niewystarczającą moc wnioskowania przy bardzo małych próbach. Tylko gdy analiza bayesowska stosowana była z użyciem wcześniejszych danych, moc wnioskowania wzrosła do zadowalającego poziomu. W końcu, wyniki naszych badań wykazały wpływ wpływ mechanicznej wentylacji po oparzeniach na przebieg objawów potraumatycznych.

Name of translator: Marcin Rzeszutek, University of Finance and Management in Warsaw, Poland

Citation: European Journal of Psychotraumatology 2015, **6**: 25216 - http://dx.doi.org/10.3402/ejpt.v6.25216
