# Supplementary material for: Analyzing small data sets using Bayesian estimation: the case of posttraumatic stress symptoms following mechanical ventilation in burn survivors
Source: Eur J Psychotraumatol. 2015 Mar 11;6:10.3402/ejpt.v6.25216. doi: 10.3402/ejpt.v6.25216 (PMC4357639; doi:10.3402/ejpt.v6.25216)
Supplement: Analyzing small data sets using Bayesian estimation: the case of posttraumatic stress symptoms following mechanical ventilation in burn survivors [file EJPT-6-25216-s002.pdf]

**Bayes Kestirimi kullanan Küçük Veri Setlerinin Analizi ve Önsel Belirlemenin Etkisi: Travma Sonrası Stres Belirtilerinin Seyri üzerinde Yanık sonrası Mekanik Ventilasyonun etkisi**

Rens van de Schoot, Joris J Broere, Koen H. Perryck, Mariëlle Zondervan-Zwijnenburg, Nancy E.E. van Loey

Özet
Arkaplan: Boylamsal çalışmalarda küçük veri setlerinin analizi güç konularına neden olabilir ve doğru olmayan (örn., yanlı) parametrik değerler konusunda sıkıntı yaratabilir. Yanık mağdurlarında mekanik ventilasyonu takip eden travma sonrası stres belirtilerine ilişkin ampirik bir örnek yoluyla gösterilmiştir. Bayes analizlerine önsel bilginin dahil edilmesi ile iki önemli konu olan güç ve yanlı parametrelerin çözülebileceğini gösterdik.Eğer bir araştırmacı Bayes  kestirimini kullanmak isterse, denilen önsel dağılımların belirlenmesi gereklidir. İlk çalışmada bu önsellerin nasıl belirlendiğini ve duyarlılık analizi yoluyla önceki (yanlış) belirlemenin tam etkisini nasıl kontrol edileceği gösterilir. İkinci bir çalışmada bir uyaran yoluyla hangi durumlarda Bayes yaklaşımının varsayılan yaklaşıma üstün geldiği gösterilir. Beklendiği şekilde, örneklem sayısı küçüldükçe sonuçların daha çok önsel belirlemelere dayandığı sonucuna ulaşılmıştır. Buna rağmen, varsayılan (örn., maksimum olasılık) tahmin çok küçük örneklemlerde  yetersiz güçle birlikte yetersiz kapsamın olduğunu göstermiştir. Bayes analizi sadece bilgi verici önsel ile birlikte kullanıldığında güç, kabul edilebilir seviyelere çıkmıştır. Sonuç olarak, veriler, mekanik ventilasyon periyodunun, yanığı takip eden travma sonrası stres bozukluğu belirtilerinin seyri üzerindeki itici etkisi hakkındaki ilk kanıtları sağlayan yanık mağdurları üzerinde analiz edilmiştir.

Anahtar Kelimeler: Bayes kestirimi, Maksimum Olasılık, Önsel belirleme, Güç, Tekrarlayan ölçüm analizi,Küçük örneklemler, yanık mağdurları, mekanik ventilasyon, TSSB

Name of Translator: Seray Akça

Citation: European Journal of Psychotraumatology 2015, **6**: 25216 - http://dx.doi.org/10.3402/ejpt.v6.25216
